# A Lightweight Model for Real-Time Detection of Vehicle Black Smoke

**DOI:** 10.3390/s23239492

**Published:** 2023-11-29

**Authors:** Ke Chen, Han Wang, Yingchao Zhai

**Affiliations:** 1 College of Surveying and Land Information Engineering, Henan Polytechnic University, Jiaozuo 454000, China; 212004010025@home.hpu.edu.cn (K.C.); 212204010020@home.hpu.edu.cn (Y.Z.); 2 School of Environment and Spatial Informatics, China University of Mining and Technology, Xuzhou 221116, China

**Keywords:** intelligent traffic, black smoke exhaust, MGSNet model, lightweight network MobileNetv3, GSConv module

## Abstract

This paper discusses the application of deep learning technology in recognizing vehicle black smoke in road traffic monitoring videos. The use of massive surveillance video data imposes higher demands on the real-time performance of vehicle black smoke detection models. The YOLOv5s model, known for its excellent single-stage object detection performance, has a complex network structure. Therefore, this study proposes a lightweight real-time detection model for vehicle black smoke, named MGSNet, based on the YOLOv5s framework. The research involved collecting road traffic monitoring video data and creating a custom dataset for vehicle black smoke detection by applying data augmentation techniques such as changing image brightness and contrast. The experiment explored three different lightweight networks, namely ShuffleNetv2, MobileNetv3 and GhostNetv1, to reconstruct the CSPDarknet53 backbone feature extraction network of YOLOv5s. Comparative experimental results indicate that reconstructing the backbone network with MobileNetv3 achieved a better balance between detection accuracy and speed. The introduction of the squeeze excitation attention mechanism and inverted residual structure from MobileNetv3 effectively reduced the complexity of black smoke feature fusion. Simultaneously, a novel convolution module, GSConv, was introduced to enhance the expression capability of black smoke features in the neck network. The combination of depthwise separable convolution and standard convolution in the module further reduced the model’s parameter count. After the improvement, the parameter count of the model is compressed to 1/6 of the YOLOv5s model. The lightweight vehicle black smoke real-time detection network, MGSNet, achieved a detection speed of 44.6 frames per second on the test set, an increase of 18.9 frames per second compared with the YOLOv5s model. The mAP@0.5 still exceeded 95%, meeting the application requirements for real-time and accurate detection of vehicle black smoke.

## 1. Introduction

The urgency of controlling exhaust pollution from motor vehicles is becoming increasingly apparent. With the development of computer vision technology, automatic detection of black smoke emissions from vehicles can be achieved using road traffic surveillance videos. Extracting features related to black smoke emissions from vehicles and combining them with classifiers can enable automatic detection of black smoke. Among the various methods, deep neural networks have been used to build object detection models that are categorized into two-stage and single-stage models [1,2,3,4]. Cao et al. [5] utilized the Inceptionv3 convolutional neural network to capture spatial information in surveillance videos with suspected black smoke frames, while a long short-term memory network learned the temporal dependencies between frames. Xia et al. [6] proposed using the LeNet-5 model, based on convolutional neural networks, for vehicle black smoke recognition, achieving an accuracy of over 80% for both positive and negative samples. By introducing the Inception module, the improved LeNet-5 model achieved a 5.38% increase in accuracy for positive sample recognition. Guo et al. [7] presented a dual-branch black smoke and vehicle detection network based on the CenterNet framework. They used vehicle feature maps to guide the training of black smoke exhaust feature maps. Test results indicated that the model achieved a detection speed of up to 25.46 FPS (frames per second) and mAP (mean average precision) of 92.5% at an intersection-over-union threshold of 0.5. Zhang et al. [8,9] used both 2D and 3D convolutions to extract spatial information and spatiotemporal features from black smoke emissions. Their approach, based on the 2D-3D multi-frame classification network, achieved a recognition accuracy of 90.3% for vehicle black smoke detection, with a single-frame image inference time of 45.9 milliseconds.

Convolutional neural networks often generate a large number of redundant feature maps when extracting image features, which is caused by repetitive calculations in traditional convolution. As the number of convolutional kernels and channels continues to increase, the model’s parameter count and computational load also grow, posing a challenge for deploying vehicle black smoke automatic detection models on mobile platforms [10,11,12]. More efficient network structure designs can effectively reduce the model’s parameter count. Wang et al. [13] proposed a lightweight network, the Yolo-Light model, for deployment on the embedded platform Jetson Nano. The recognition accuracy for the vehicle black smoke test set was 91.57%, with a detection speed of 16 FPS. Zhang et al. [14] designed lightweight networks, YOLOv3-M3-CBAM and YOLOv4-GhostNet, based on YOLOv3 and YOLOv4 models; the improved models achieved a detection speed of 20 FPS. Du et al. [15] introduced a lightweight network, the RA-YOLOv5s model, which incorporates spatial and channel attention mechanisms in CSP modules to learn useful image features. Zhao et al. [16] addressed the issue of poor real-time performance in table-tennis-ball object detection by proposing a lightweight improved network, the SYOLO5 model, which compresses the model’s parameter count to one-fifth of the original model. The improved lightweight network, ShuffleNetv2, restructured the YOLOv5 model’s backbone network and introduced an efficient attention mechanism in the feature fusion process to enhance detection performance. Peng et al. [17] presented a lightweight network, the Ghost-YOLOv5-Shuffle model, which uses the GhostBottleneckCSP module and ShuffleConv module with fewer parameters to replace the C3 module in the YOLOv5 model’s backbone network, reducing the computational load in the feature channel fusion process.

Existing vehicle black smoke automatic detection models face challenges related to high parameter counts and computational load, making their deployment on mobile platforms difficult. However, lightweight model improvement solutions have not effectively balanced the accuracy of vehicle black smoke recognition with the demands of real-time detection applications. Therefore, this study, based on the excellent object detection performance of the YOLO network framework, proposes a lightweight real-time vehicle black smoke detection network called MGSNet. The experiments involved the reconstruction of the YOLOv5s model’s backbone feature extraction network using three different lightweight networks: ShuffleNetv2, MobileNetv3, and GhostNetv1. The accuracy and speed evaluation metrics of the models before and after lightweight improvements were compared to determine the best lightweight backbone network improvement solution. A new convolution module called GSConv is introduced into the neck network of the YOLOv5s model to enhance black smoke feature expression. This allows the lightweight improved vehicle black smoke real-time detection network, MGSNet, to be suitable for small-capacity embedded devices.

## 2. YOLOv5 Network Framework

The YOLO series algorithms for single-stage object detection can significantly improve detection speed while sacrificing only a small amount of accuracy. Therefore, this paper selects the YOLOv5 model, known for its excellent object detection performance, as the foundational network framework for lightweight improvement. Different versions of the YOLOv5 model are available, varying in terms of network depth and width, including sizes such as “s,” “m,” “l,” and “x,” and also come in versions like 1.0, 5.0, and 6.0. Taking into consideration the relatively small size of the homemade vehicle black smoke dataset used in this study and the real-time detection requirements, the decision was made to choose YOLOv5s version 6.0 as the base network for lightweight improvements [18,19]. The YOLOv5s model is structured in four main parts: the input layer, the backbone network, the neck network, and the output layer. The input layer involves image preprocessing, including mosaic data augmentation, adaptive image scaling, and adaptive anchor box adjustments. The output layer is responsible for detecting the categories and positions of target objects and is primarily composed of loss functions and non-maximum suppression techniques [20,21].

The backbone network is primarily composed of modules including the Conv (convolution) module, C3 module, and SPPF (spatial pyramid pooling fast) module. In version 6.0, the original focus module is replaced with a convolutional layer with a convolution kernel of 6, a stride of 2, and a padding of 2. This change was made to improve efficiency, especially for GPUs with limited performance. Using a convolutional layer in this context is more efficient than the focus module [22]. Version 6.0 employs the C3 module, which serves a similar role as the CSP module but differs in that it eliminates the ordinary convolution module after removing the residual output. The neck network utilizes a feature pyramid network combined with a path aggregation network structure to further process features extracted at different stages. The feature pyramid network employs upsampling to convey stronger semantic information from deeper feature maps to shallower ones, while the path aggregation network uses downsampling to transmit positional information from shallower feature maps to deeper ones. This simultaneous up and down sampling achieves multi-scale feature fusion [23,24].

## 3. Lightweight Model Improvements

### 3.1. Backbone Network Lightweight Optimization

**Figure 1:**
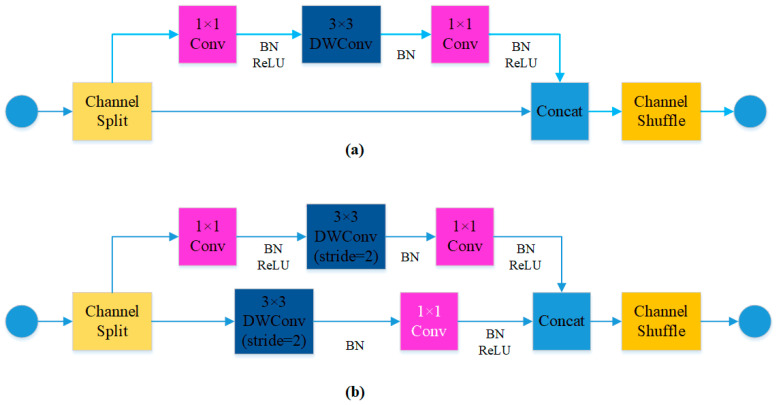
Shuffle module structure diagram: (**a**) stride = 1; (**b**) stride = 2.

The lightweight network ShuffleNetv2’s structural design takes into account the memory access cost of different network structures and proposes a more efficient shuffle module. It introduces the channel split operation, which divides the input feature map into two branches along the channel dimension. The results of these two branches are then connected, followed by a channel shuffle operation to merge the channel information [25,26]. In this study, the YOLOv5s model’s backbone feature extraction network, CSPDarkNet53, has been restructured using the lightweight ShuffleNetv2 network. A total of six shuffle modules are employed. The first shuffle module is preceded by a CBRM consisting of Conv, BN, ReLU, and Maxpool. The CBS module, which reconstructs the original SPPF module, is placed before the output of the backbone network and consists of Conv2d, BN, and SiLU. Figure 1b corresponds to the shuffle module with a stride of 2, directly inputting the feature map into two branches, each containing depthwise separable convolutions with a stride of 2. The feature maps are then connected, followed by the channel shuffle operation.

**Figure 2:**
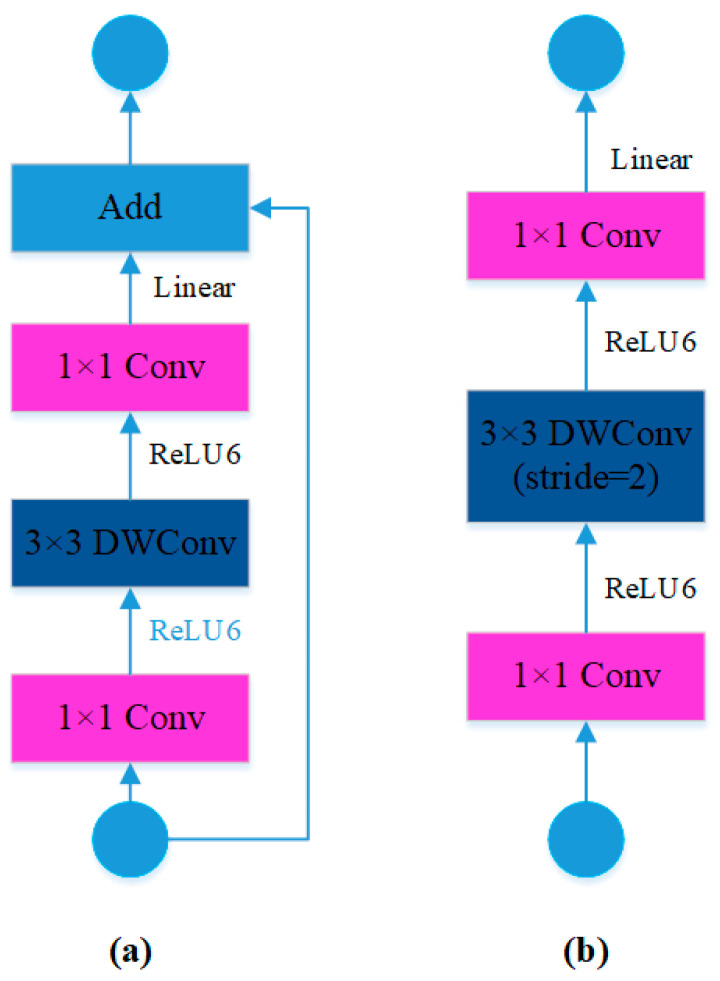
Inverted residual structure diagram: (**a**) stride = 1; (**b**) stride = 2.

The lightweight MobileNetv3 not only continues the depthwise separable convolution from the v1 version but also maintains the inverted residual structure introduced in the v2 version. The novel aspect of its design is the incorporation of the SE attention mechanism, which consists of compression and excitation parts [27,28]. After global average pooling, features are sequentially input into two fully connected layers with ReLU6 and h-swish activation functions. The h-swish activation function effectively addresses the issue of complex derivative computation [29]. Taking into account the relatively small custom vehicle black smoke dataset used in this study and the requirement for real-time detection, the study chooses the lightweight MobileNetv3-small to restructure the CSPDarkNet53 backbone feature extraction network of the YOLOv5s model. A total of eleven MB modules have been used for this reconstruction. The first MB module is preceded by a CBH module. Depending on the stride, the inverted residual structure can be categorized into the two cases illustrated in Figure 2.

**Figure 3:**
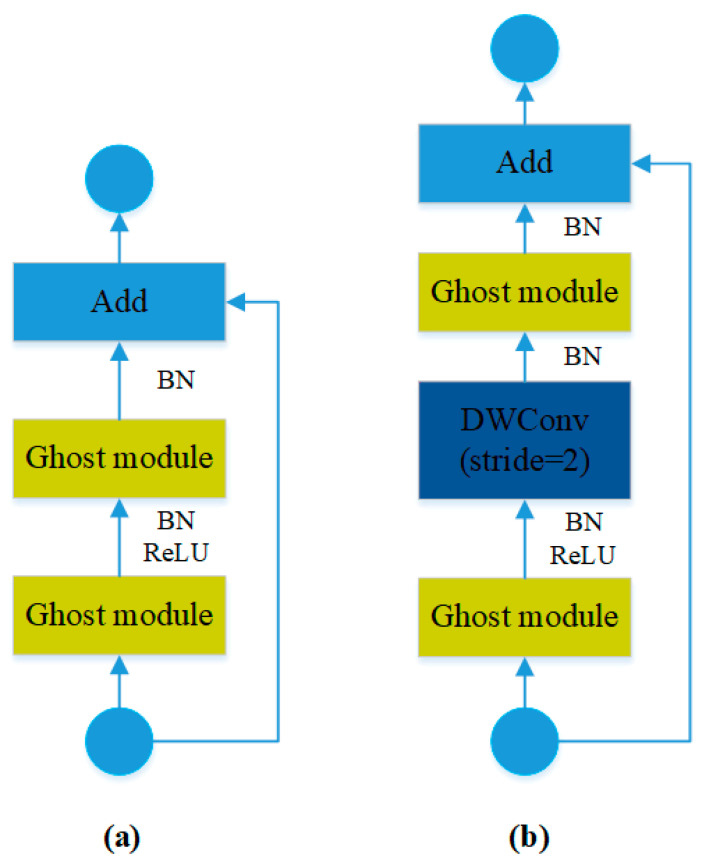
Ghost bottlenecks structure diagram: (**a**) stride = 1; (**b**) stride = 2.

The novelty in the structural design of the lightweight GhostNetv1 network lies in the introduction of a new Ghost module, which condenses essential features from the input by using a small number of regular convolutions. Initially, necessary features are condensed through a series of simple linear operations, generating redundant feature layers. Finally, the feature layers generated by convolutions are concatenated with the redundant feature layers generated by linear operations [30]. The Ghost module combines the redundant features it extracts with the features obtained through simple linear operations, resulting in a large number of black smoke feature maps with lower computational load for the simple linear operations. In this study, the lightweight GhostNetv1 network has been used to restructure the CSPDarkNet53 backbone feature extraction network of the YOLOv5s model while retaining the original SPPF module. The SPPF module can fuse multiple receptive fields and separate the most significant contextual features. This allows it to extract spatial feature information of different sizes, enhancing the model’s robustness [31]. When the stride is 1, Ghost bottlenecks consist of two directly stacked Ghost modules, and the second Ghost module does not use an activation function when producing its output. When the stride is 2, depthwise separable convolution layers are introduced to compress the width and height dimensions, as shown in Figure 3.

### 3.2. Neck Network Lightweight Optimization

**Figure 4:**
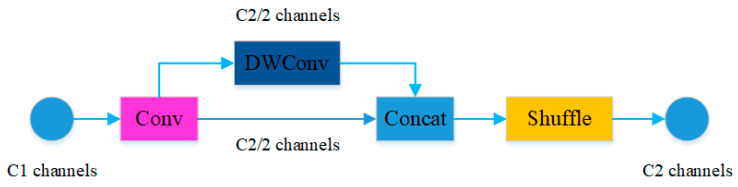
Convolution module GSConv structure diagram.

The neck network introduces a new convolution module called GSConv to restructure the standard convolution module Conv, as shown in Figure 4. The GSConv module strives to retain these connections with lower computational complexity. It combines regular convolution with depthwise separable convolution followed by channel shuffling to maintain accuracy while reducing the model’s computational load to the maximum extent [32]. The channel shuffling operation merges information generated by regular convolution with information produced by depthwise separable convolution, achieving a better balance between model accuracy and speed. Channel-dense convolutions maximize the interconnections between each channel, whereas channel-sparse convolutions completely sever these connections [33]. The GSConv module enhances the network’s non-linear expressive capability by adding depthwise separable convolution layers and channel shuffling operations. Experiments have shown that introducing the GSConv module to replace regular Conv in various stages of the model results in a deeper network [34]. When the feature maps extracted by the YOLOv5s model’s backbone network are input to the neck network, their channel size has reached its maximum value, while the width and height dimensions are at their minimum. Therefore, the neck network introduces the GSConv module to process the input feature maps, reducing redundant and repetitive information in the network.

### 3.3. MGSNet Model

**Figure 5:**
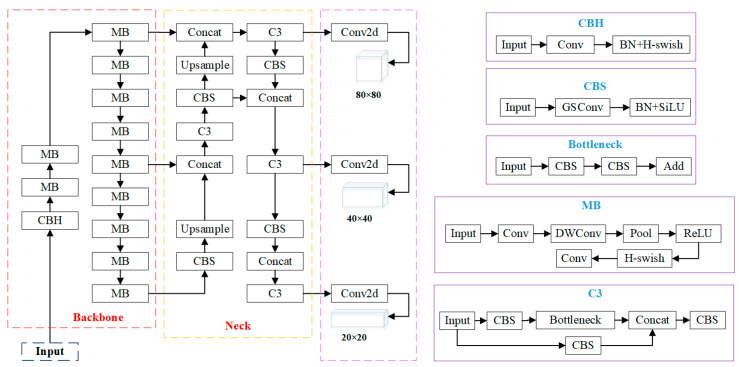
The network architecture diagram of the MGSNet model.

This study is based on the YOLOv5s network framework and focuses on lightweight improvements to the backbone feature extraction network and the neck network to increase network inference speed by reducing the model’s parameter count and computational load. Various existing lightweight networks have their strengths in network structure design. The experiment involved the reconstruction of the CSPDarknet53 backbone feature extraction network using three different lightweight networks: ShuffleNetv2 [35], MobileNetv3 [36], and GhostNetv1 [37]. A comprehensive comparison of accuracy and speed evaluation metrics was conducted. Meanwhile, a new convolution module called GSConv (group shuffle convolution) [38] was introduced in the neck network to restructure the standard convolution module Conv. Deconstruction experiments were designed to validate the lightweight improvement effects of the GSConv module. Figure 5 illustrates the structural framework of the lightweight real-time vehicle black smoke detection network, MGSNet, proposed in this study based on the YOLOv5s network framework. It first employs MobileNetv3 to reconstruct the CSPDarkNet53 backbone network of the YOLOv5s model. Additionally, the neck network introduces a new convolution module, GSConv, to replace the standard Conv. The lightweight MobileNetv3 network introduces a lightweight SE (squeeze and excitation) attention module that better extracts black smoke feature information. The inverted residual structure facilitates the flow of black smoke feature information between layers, and the h-swish activation function accelerates computation [39,40]. The GSConv module combines depthwise separable convolution with standard convolution. The depthwise separable convolution reduces the model’s computational complexity, while standard convolution alleviates issues related to weak feature fusion capabilities in depthwise separable convolution, which can result in lower recognition accuracy [41,42,43].

## 4. Experimental Preparation

### 4.1. Experimental Environment and Parameter Configuration

The experimental environment configuration parameters, as shown in Table 1, were set based on previous relevant research and comparative experiments. The resolution of input images was uniformly scaled to 640 × 640. Prior to training, initial anchor boxes were clustered using the k-means algorithm, resulting in (10, 13, 16, 30, 33, 23), (30, 61, 62, 45, 59, 119), and (116, 90, 156, 198, 373, 326). The model was trained for a total of 200 epochs with a batch size of eight. The optimizer chosen was the Adam algorithm, with an initial learning rate of 1 × 10^−3^ and an initial decay rate of 1 × 10^−5^. Learning rate reduction was achieved through the cosine annealing strategy. Random dropout techniques were applied to the fully connected layers to prevent overfitting during model training, with a dropout rate of 0.5 [44,45,46].

### 4.2. Custom Dataset

**Figure 6:**
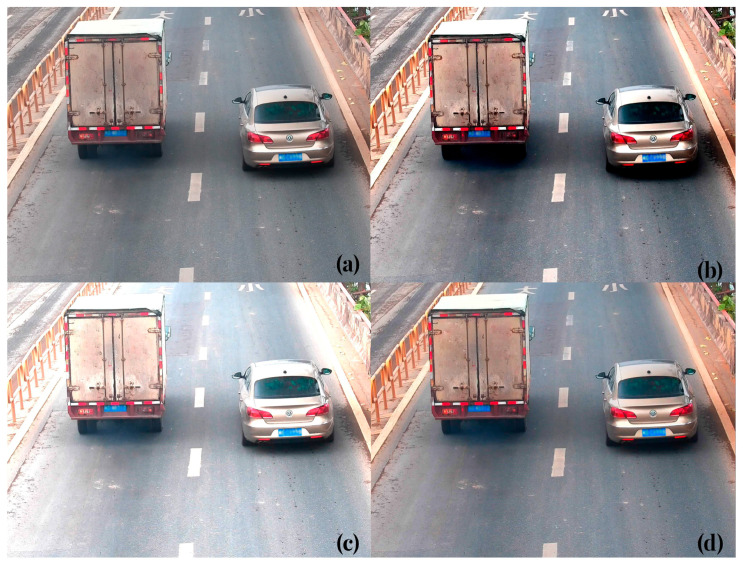
Effect images of different data augmentation methods: (**a**) original image; (**b**) contrast enhancement; (**c**) brightness enhancement; (**d**) color enhancement.

The study aim is to perform real-time detection of whether motor vehicles on the road are emitting black smoke emissions, with research data sourced from road traffic monitoring videos. The color characteristics of the target black smoke emissions can be further explored by enriching the custom vehicle black smoke dataset through data augmentation methods such as adjusting image brightness and contrast. In the experiment, the LabelImg software (version 4.5.13) was used to annotate black smoke emissions. The dataset consisted of 2362 images of vehicle black smoke data, which were divided into a training set, a test set, and a validation set in an 8:1:1 ratio. Sample datasets before and after data augmentation are illustrated in Figure 6; Figure 6a represents the original image from the road monitoring video, while Figure 6b–d represent images processed with contrast enhancement, brightness enhancement, and color enhancement, respectively. The contrast enhancement can expand the contrast of the features of interest, while brightness and color enhancement play a role in image clarity and color distinction. Using data augmentation techniques related to color attributes not only enriches the custom vehicle black smoke dataset but also extends the image features of the areas of interest where black smoke emissions are present.

### 4.3. Evaluation Metrics

The confusion matrix is a metric for assessing the results of a model, helping to determine the quality of model classification. It is also known as an error matrix. Based on the results of the confusion matrix, various secondary evaluation metrics can be calculated, such as *Precision*, *Recall*, *F1_Score*, and *mAP* (mean average precision), where mAP@0.5 [47,48] refers to the mean average precision at an intersection over union threshold of 0.5.

(1) $$P=\frac{TP}{TP+FP}$$

(2) $$R=\frac{TP}{TP+FN}$$

(3) $$AP=\int_{0}^{1}PR\mathrm{dr}$$

(4) $$mAP=\frac{1}{n}\sum_{i=0}^{1}AP_{i}$$

(5) $$F1\_score=2\frac{PR}{P+R}\times 100\%$$

In the equation, TP represents the number of true positive samples, FP represents the number of false positive samples, and FN represents the number of false negative samples. The index i denotes different classes, and in this study, it specifically refers to black smoke emissions. The value of model parameters indicates the amount of memory space required for model computation, with smaller values suggesting less memory usage. The FPS represents the number of images the model can process per second, and a higher value indicates better real-time performance for model computation.

## 5. Experimental Results and Analysis

### 5.1. Performance Comparison of Lightweight Networks

In this study, three different lightweight networks, ShuffleNetv2, MobileNetv3, and GhostNetv1, were used, respectively, to restructure the backbone feature extraction network (CSPDarknet53) of the YOLOv5s model. Experimental comparisons were conducted to assess the performance differences before and after the improvements. Table 2 presents the test results for the models after the improvements using different lightweight networks. YOLOv5s-S represents the lightweight model with the main feature extraction network restructured using ShuffleNetv2; YOLOv5s-M represents the lightweight model with the main network replaced by MobileNetv3; YOLOv5s-G represents the lightweight model with the main network restructured using GhostNetv1.

Comparing the precision evaluation metrics of the models, YOLOv5s-G shows a slight improvement in all precision metrics, while YOLOv5s-S exhibits a decreasing trend in all metrics. YOLOv5s-M has a 1.1% increase in precision but a 3.3% decrease in recall compared with YOLOv5s. In terms of speed evaluation metrics, all three different lightweight network improvements reduce the model’s parameter count and computational complexity. YOLOv5s-S, in particular, compresses the parameter count to well below one million, representing only 1/10 of the original model’s parameters. YOLOv5s-M also reduces the parameter count to 1/6 of the original model. YOLOv5s-S exhibits a notable advantage in inference speed, while YOLOv5s-G surpasses the original in detection precision. However, YOLOv5s-M strikes a better balance between model precision and speed, ensuring both accuracy and lightweight model design. Its inference speed meets the real-time requirements for vehicle black smoke detection. As a result, this study ultimately chose to restructure the backbone feature extraction network CSPDarknet53 of YOLOv5s using MobileNetv3 for lightweight improvement.

### 5.2. Ablation Experiments

The neck network of the YOLOv5s model is primarily composed of two upsampling modules, four CBS modules, and four C3 modules, where both the CBS and C3 modules contain standard Conv convolution layers. In this study, a new convolution module, GSConv, was introduced to replace the standard Conv convolution modules, and a set of ablation experiments were conducted to verify the lightweight improvement of the new GSConv module. The results of the ablation experiments are presented in Table 3. In the table, “YOLOv5s-GS-CBS” denotes the introduction of GSConv to restructure the Conv in the CBS modules of the neck network, “YOLOv5s-GS-C3” represents the introduction of GSConv to restructure the Conv in the C3 modules, “YOLOv5s-GS” means the introduction of GSConv to restructure all the Conv modules in the neck network of the YOLOv5s model, and “MGSNet” signifies the introduction of GSConv to restructure all the Conv modules in the neck network of the YOLOv5s-M lightweight model. Introducing GSConv in the CBS and C3 modules in the neck network effectively reduced the parameter count of the YOLOv5s model. Particularly, replacing Conv with GSConv in the C3 modules yielded better results, reducing the model’s parameter count by 2.7%. Therefore, the introduction of the new convolution module GSConv restructures all the regular convolution modules in the neck network of the lightweight YOLOv5s-M model. The real-time vehicle smoke detection network after lightweight improvement is referred to as MGSNet.

Comparing the evaluation metrics before and after the lightweight model improvement, it is evident that the precision of MGSNet slightly decreases after lightweight improvement. Specifically, precision, recall, mAP@0.5, and F1_score decrease by 1%, 2.1%, 2.9%, and 2% respectively, in comparison to the YOLOv5s model. However, the mAP@0.5 value still reaches 95.4%. After lightweight improvement, MGSNet experienced a significant reduction in model parameters and computational complexity. The parameter count has been compressed to 1/6 of the original model, and the number of images processed per second increased from 25 images to 44 images. The results of the ablation experiments demonstrate the feasibility of the lightweight improvement proposed in this study, as it can significantly reduce model parameters and computational complexity.

**Figure 7:**
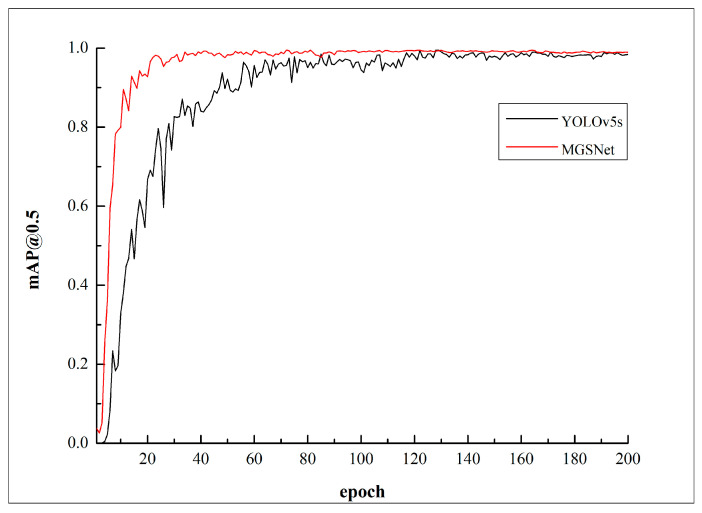
mAP@0.5 change curve graph before and after lightweight improvements.

**Figure 8:**
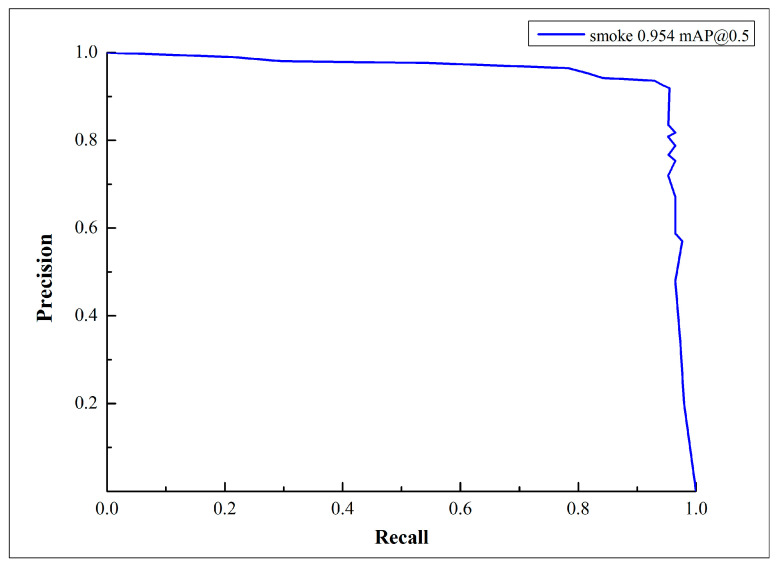
P-R curve graph for the MGSNet model training.

The change curve of mAP@0.5 before and after lightweight improvements is shown in Figure 7; the red curve represents the MGSNet model, and the black curve represents the YOLOv5s model. The model was trained for 200 epochs, and after about 50 epochs of training, the mAP@0.5 value of the MGSNet model reached around 0.95 with minimal fluctuations. The model exhibited faster learning efficiency, and its accuracy remained almost unchanged before and after the lightweight improvements. The change in the P-R curve during the training of the MGSNet model is shown in Figure 8, with the horizontal axis representing the recall rate and the vertical axis representing the precision rate. Precision decreases as recall rate increases, and the area enclosed by the P-R curve corresponds to the mAP@0.5 value.

### 5.3. Comparison of Existing Algorithms

**Figure 9:**
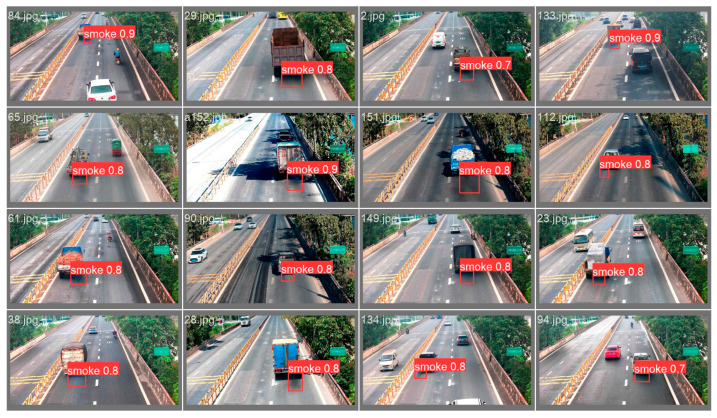
Test results of the MGSNet model.

To demonstrate the real-time detection advantage of the lightweight improvement model MGSNet proposed in this study, experiments compared the MGSNet model with the YOLOv5s model and other lightweight improvement models based on the YOLOv5s network framework using our custom vehicle smoke dataset. The test results of different models are shown in Table 4. The RA-YOLOv5s model focuses on lightweight improvements to the residual units of the YOLOv5s backbone CSP module. The test results indicate that the model’s parameter count was reduced by only 5%, while there was a significant decrease in detection accuracy. The SYOLO5 model involves the reconstruction of the YOLOv5 model’s backbone CSPDarkNet53 using the lightweight network ShuffleNetv2, compressing the model’s parameter count to 1/5 of the YOLOv5s model. The inference time for a single image is 23.6 ms, and the lightweight improvement effect is more noticeable. The Ghost-YOLOv5-Shuffle model is capable of significantly reducing the model’s parameter count, thanks to the replacement of original modules with lightweight Ghost and shuffle modules. Figure 9 displays real-time detection results of the lightweight improvement model MGSNet proposed in this study, accurately identifying vehicle smoke in the video.

## 6. Conclusions

This study proposes a lightweight real-time detection model for black-smoke-emitting vehicles, named MGSNet, based on the YOLOv5s framework. The goal is to meet the requirements of real-time detection of black-smoke-emitting vehicles and reduce the deployment complexity for mobile applications. The approach involves restructuring the main feature extraction network CSPDarkNet53 using the lightweight MobileNetv3 for YOLOv5s, and introducing a novel convolution module, GSConv, to restructure all standard Conv modules in the neck network. The experimental results demonstrate that the lightweight MGSNet model achieved significant reductions in parameter count, down to 1/6 of the YOLOv5s model’s parameters. The inference time for a single image has been reduced by 16.5 ms. The model can process 44 images per second, meeting the real-time requirements for automatic detection of black-smoke-emitting vehicles. MGSNet exhibited excellent performance on the test set, with a precision of 95.7%, recall of 94.7%, mAP@0.5 of 95.4%, and an F1 score of 95%. During testing, it was observed that black smoke emissions occupy a small portion of the entire image. Future research will focus on optimizing the network structure to ensure detection accuracy for small objects, while maintaining the improved inference speed of the MGSNet model.

## Figures and Tables

**Table 1:** Experimental environment configuration.

Name	Version Model
Operating system	Windows10
CPU	Intel(R) Core (TM) i5-11400F @2.60 GHz
GPU	NVIDIA GeForce GTX 1650
Programming language	Python3.8.13
Deep learning framework	Pytorch1.13.0, CUDA11.7

**Table 2:** Test results of different lightweight network improvements.

Model	P (%)	R (%)	mAP (%)	F1_Score (%)	Parameters (M)	FPS
YOLOv5s	0.967	0.968	0.983	0.97	7012822	25.7
YOLOv5s-S	0.955	0.947	0.955	0.94	842358	52.0
**YOLOv5s-M**	**0.978**	**0.935**	**0.967**	**0.96**	**1354454**	**37.6**
YOLOv5s-G	0.969	0.988	0.987	0.98	5078974	31.5

**Table 3:** Results of ablation experiments.

Model	P (%)	R (%)	mAP (%)	F1_Score (%)	Parameters (M)	FPS
YOLOv5s-GS-CBS	0.963	0.962	0.981	0.97	6905846	27.0
YOLOv5s-GS-C3	0.958	0.960	0.980	0.96	6825896	27.6
YOLOv5s-GS	0.961	0.959	0.978	0.96	6764322	28.5
**MGSNet**	**0.957**	**0.947**	**0.954**	**0.95**	**1280204**	**44.6**

**Table 4:** Comparison of different lightweight improvement models.

Model	P (%)	mAP (%)	Parameters (M)	FPS
YOLOv5s	0.967	0.983	7012822	25.7
RA-YOLOv5s [15]	0.907	0.916	6658660	27.5
SYOLO5 [16]	0.942	0.932	1380947	42.4
Ghost-YOLOv5-Shuffle [17]	0.936	0.938	2419191	34.6
**MGSNet**	**0.957**	**0.954**	**1280204**	**44.6**

## Data Availability

The data presented in this study are not publicly available due to privacy restrictions.
